# Immune Thrombocytopenia After COVID-19 Vaccine Requiring Splenectomy: A Case Report and Review of the Literature

**DOI:** 10.7759/cureus.53955

**Published:** 2024-02-10

**Authors:** Lara Alkhelaiwy, Jumana A Fatani, Ismaeil Alhamoud, Ahmed Chaballout

**Affiliations:** 1 General Surgery, Specialized Medical Center, Riyadh, SAU; 2 General Surgery/Kidney Transplant and Vascular Surgery, Specialized Medical Center, Riyadh, SAU

**Keywords:** immune thrombocytopenia, thrombocytopenia, itp, splenectomy, vaccine, covid-19

## Abstract

Post-vaccination immune thrombocytopenia (ITP) is a rare but recognized adverse event believed to result from an autoimmune reaction triggered by the vaccine. This case report presents the fourth documented instance of severe ITP requiring splenectomy following the administration of a COVID-19 vaccine. The patient, a 54-year-old previously healthy female with no familial history of autoimmune or hematological disorders, developed ITP two weeks after the first dose of the COVID-19 vaccine. While most ITP cases associated with COVID-19 vaccines manifested after the second dose, this unique case demonstrated symptoms following the initial vaccination. Initially responsive to first-line management, the patient experienced a relapse upon receiving the second dose from a different vaccine manufacturer. Despite exhaustive medical interventions, the refractory nature of the condition persisted, ultimately mandating splenectomy for the achievement of complete remission. This case underscores the potential for serious, refractory ITP with the second dose of a COVID-19 vaccine, particularly in patients who initially developed ITP after the first dose, even if they had seemingly achieved complete remission. These findings emphasize the importance of vigilant monitoring and individualized treatment strategies in such cases, contributing valuable insights to the growing body of knowledge surrounding vaccine-induced ITP.

## Introduction

Immune thrombocytopenia (ITP) is an autoimmune disorder characterized by platelet counts below 100 K/uL, normal bone marrow, and no other identifiable causes of thrombocytopenia. In ITP, platelets have a shortened half-life due to anti-platelet antibodies [1]. In the context of vaccinations, post-vaccination ITP is a rare but recognized adverse event believed to result from an autoimmune reaction triggered by the vaccine. The risk of post-vaccination ITP is higher after certain vaccines, such as the measles, mumps, and rubella (MMR) vaccine and the varicella vaccine [2]. COVID-19 vaccines, such as those based on messenger RNA (mRNA) technology, instruct the body's cells to produce a protein that stimulates an immune response against the SARS-CoV-2 virus. Cases of post-vaccine ITP have been reported after both mRNA COVID-19 vaccines (Pfizer-BioNTech and Moderna) and the AstraZeneca COVID-19 vaccine [2]. While most cases of post-vaccine ITP are mild and can be managed with supportive care [3,4]. We present a unique case of severe and refractory ITP requiring intensive treatment, including splenectomy. As of date, there have been only three cases in the literature of ITP post COVID-19 vaccine requiring splenectomy [5-7]. We present a case of a patient who developed severe refractory ITP two weeks after receiving the COVID-19 vaccine. The patient required a splenectomy to achieve sustained remission.

## Case presentation

A 54-year-old Filipino female with no prior medical or surgical history, known allergies, or family history of hematologic diseases, immunodeficiency, or malignancy presented to our clinic. She had entered menopause two years prior to the onset of her symptoms.

The patient's symptoms began approximately two weeks after receiving the first dose of the Pfizer COVID-19 vaccine. She reported frequent and heavy episodes of epistaxis during this period. Notably, she had no history of spontaneous bleeding or excessive bruising and denied any gastrointestinal or urinary tract bleeding. Importantly, the patient was not taking any medications at the time. Upon physical examination, no signs of active bleeding, hepatosplenomegaly, or petechiae were observed.

Laboratory investigations revealed a significantly low platelet count of 20 K/uL, prompting her admission for further assessment and management of severe thrombocytopenia. A peripheral blood smear displayed the presence of giant platelets, a potential indicator of ITP, with normal red and white cell morphology. Additionally, her lactate dehydrogenase levels were notably elevated at 508 U/l (normal range: 135-214), possibly indicating tissue injury associated with severe thrombocytopenia. All other laboratory parameters, including hemoglobin levels, leukocyte counts, coagulation profiles, renal and liver function tests, inflammatory markers, and antinuclear antibodies (ANA) results, were within normal limits. Furthermore, viral screenings for hepatitis B and C and HIV yielded negative results.

Given the significantly low platelet count, the presence of giant platelets on blood smears, and the exclusion of alternative causes, a provisional diagnosis of ITP was strongly suspected. The patient was initiated on methylprednisolone therapy and received platelet transfusions, resulting in an improvement in her platelet count to 87 K/uL. She was discharged and remained asymptomatic during the subsequent four months, receiving regular follow-up care and monitoring. However, approximately 15 days after the second Moderna COVID-19 vaccine dose, the patient experienced recurrent epistaxis and dizziness. Hospitalization and dexamethasone therapy brought her platelet count to 69 K/uL at discharge, followed by outpatient follow-up and oral steroids. Over the next four months, despite ongoing oral steroids, she suffered multiple admissions with severe thrombocytopenia (platelets as low as 3 K/uL) and recurrent, severe epistaxis requiring nasal packing. Tranexamic acid during epistaxis episodes controlled the bleeding. Pulsed corticosteroids initially increased platelets to 56 K/uL, but the effect was transient, and steroids became ineffective. Eltrombopag (a thrombopoietin receptor agonist) for 48 days failed to raise her platelet count. High-dose intravenous immunoglobulin (IVIG) initially boosted it to 246 K/uL, but subsequent infusions proved ineffective. Weekly rituximab infusions initially increased her platelets to 51 K/uL, but the effect waned, and her count dropped to 1 K/uL despite receiving four cycles, along with high-dose IVIG, high-dose steroids, and ongoing thrombopoietin receptor agonist (TPO-RA) therapy. With all other options exhausted and her platelet count critically low, a splenectomy was planned as the next course of action.

**Figure 1:**
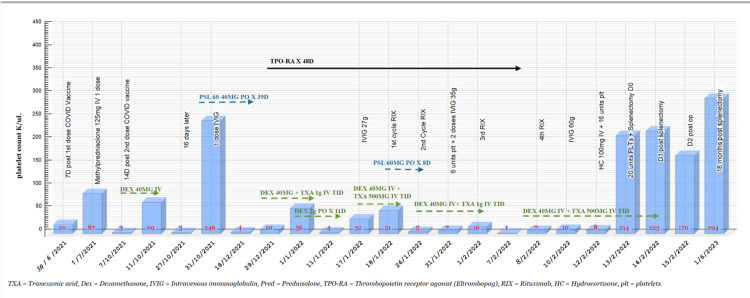
The patient’s treatment course and platelet count.

The patient had received the *Haemophilus influenzae*, meningococcal, and pneumococcal (PSV13) vaccine one month before the surgery. The preoperative manual platelet count was 8 K/uL, and she received 16 units of platelets preoperatively and 20 units in the operating room. The laparoscopic splenectomy was successfully performed without complications, and intraoperative findings revealed a normal spleen with no gross abnormalities or active infection. Post surgery, her platelet counts rapidly increased from 17 K/uL immediately after the procedure to 223 K/uL on day one postoperatively. Despite a minor decrease to 170 K/uL on day two, which is within the expected range of post-surgical fluctuations, the overall trend was a substantial increase in platelet count. She was discharged in good condition on day four with instructions for regular monitoring and platelet count checks. Figure 1 demonstrates the patient’s medications and platelet count over the treatment course.

Histopathological examination of the spleen revealed splenic congestion and prominent extramedullary hematopoiesis, findings sometimes associated with ITP and potentially contributing to impaired platelet production or increased destruction. No malignancy was identified. Remarkably, the patient achieved complete remission following laparoscopic splenectomy, with her latest platelet count at 294 K/uL one year after the operation. She continues to receive regular follow-up care and currently does not require any additional medications.

## Discussion

Although rare, vaccine-induced ITP is acknowledged as a potential side effect of several vaccines. These include MMR, varicella, inactivated hepatitis B, diphtheria-tetanus-acellular pertussis (DTaP), pneumococcus, *Haemophilus influenzae* B, varicella zoster virus (VZV), human papillomavirus (HPV), and polio vaccines [8]. The exact mechanism leading to ITP following COVID-19 vaccination remains unclear, but the vaccination itself is considered a potential contributor [9]. In this case, the diagnosis was prompted by episodes of epistaxis. Other potential causes of reduced platelet count, such as connective tissue diseases, lymphocytic leukemia, hepatitis, and human immunodeficiency virus, were ruled out, pointing to COVID-19 vaccination as the likely instigating factor. The precise process by which the vaccine might initiate an immune response, resulting in either the destruction of platelets or impaired platelet production, is not fully understood. However, it could involve mechanisms such as molecular mimicry or the stimulation of autoantibody production. This, in turn, activates autoreactive B or T lymphocytes, leading to the emergence of antiplatelet antibodies, epitope spreading, a polyclonal immune reaction, and ultimately the manifestation of ITP [2]. Many cases of vaccine-induced ITP are mild and self-limiting, and none have experienced a recurrence following the administration of the second MMR dose [3].

Since the onset of the COVID-19 pandemic and the introduction of the COVID-19 vaccine, numerous case reports have emerged regarding COVID-19-induced ITP [9]. However, a considerable number of these cases have shown positive responses to medical interventions [2,4]. To date, only three cases have been documented in the existing literature where ITP developed after COVID-19, resisted all medical treatments, necessitated splenectomy, and ultimately attained complete remission [5-7]. Table 1 summarizes the reported cases in the literature. None of the documented cases had pre-existing ITP, although two patients had a family history of autoimmune disease or ITP in a family member [5,7]. This suggests a possible genetic predisposition to developing ITP. However, familial predisposition may not be a consistent risk factor for vaccine-induced ITP, as our case did not have a family history of any autoimmune diseases. Unlike the previously reported cases where symptoms emerged one to two weeks after the second dose of the COVID-19 vaccine [5-7], our case experienced symptoms one week after the first dose and responded well to initial ITP management.

**Table 1:** Reported cases of severe refractory ITP post COVID-19 vaccine requiring splenectomy. ITP: immune thrombocytopenia; TPO-RA: thrombopoietin receptor agonist; IVIG: intravenous immunoglobulin.

Article	Age	Gender	Medical history	Family history	Onset of symptoms	Vaccine manufacturer	Platelets count at the time of presentation	Symptoms	Medical treatments	Platelets transfusion	Platelets level pre-op	Splenectomy	Platelets count after splenectomy
Ito et al. (2022) [6]	50 years old	Female	None	None	2 weeks after the 2nd dose	Pfizer	1 K/uL	Menorrhagia	Steroids, TPO-RA, IVIG	Yes	106 K/uL after platelets transfusion	After 9 weeks	75 K/uL
Weiner et al. (2022) [5]	42 years old	Male	Asthma	Scleroderma	1 week after the 2nd dose	Moderna	2 K/uL	Petechiae, hematoma, epistaxis	Steroids, IVIG, TPO-RA, dapsone, tranexamic acid, vincristine, rituximab	No	181 K/uL after IVIG	After 80 days	302 K/uL
Koilpillai et al. (2022) [7]	20 years old	Male	Obesity	Refractory ITP	2 weeks after the 2nd dose	Pfizer	1 K/uL	Petechiae	Steroids, IVIG, rituximab, TPO-RA, plasmapheresis, ciclosporin	Yes	2-7 K/uL	After 40 days	300 K/uL
Our case	54 years old	Female	None	None	2 weeks after the 1st dose	1st dose Pfizer, 2nd dose Moderna	20 K/uL	Epistaxis	Steroids, tranexamic acid, IVIG, TPO-RA, rituximab	Yes	214 K/uL	After 7 months	294 K/uL

In contrast to the MMR vaccine, which does not typically lead to recurrence after the second dose [3], our case developed severe refractory ITP after receiving the second dose of the COVID-19 vaccine. The decision to proceed with splenectomy was made following an extensive trial of medical therapies, aligning with the American Society of Hematology guidelines for ITP treatment. Approximately 80% of patients with ITP experience an immediate increase in platelet counts post splenectomy, and durable remission is achieved in 50% to 70% of cases [10].

Careful consideration of the risks, including potential postoperative complications and heightened susceptibility to infections, is essential when deciding to undergo a splenectomy. Preoperative management is crucial for this procedure [10]. Ideally, it is recommended to achieve platelet counts above 50 K/μL before the surgery. In this case, preoperative measures involved the administration of steroids, tranexamic acid, IVIG, and a platelet transfusion of 16 units preoperatively [11]. To prevent severe infections post splenectomy, it is imperative to implement effective infection prevention strategies. In this context, vaccination against *Haemophilus influenzae*, meningococcal, and pneumococcal (PSV13) was conducted one month before the splenectomy [10].

This case exemplifies the need for a nuanced approach to ITP management post COVID-19 vaccination, emphasizing the potential for severe and refractory cases. It encourages vigilance and proactive management in patients presenting with thrombocytopenia post vaccination, considering both medical and surgical options. The long-term remission achieved in this case demonstrates the efficacy of splenectomy in vaccine-induced refractory ITP, providing an important reference point for clinicians managing similar cases. Further research into the pathophysiology of vaccine-induced ITP and the long-term outcomes of splenectomy in this context is warranted to optimize patient care.

## Conclusions

This case report adds to the emerging knowledge of severe and refractory ITP following COVID-19 vaccination, with our patient representing only the fourth documented instance requiring splenectomy. The successful outcome highlights the value of prompt diagnosis and individualized management in such cases, considering both medical and surgical options. Recognizing the potential for ITP, particularly after the first dose, emphasizes the importance of vigilant monitoring and proactive intervention in patients experiencing post-vaccination bleeding. By contributing to understanding the spectrum of potential complications, this case provides valuable insights for optimizing patient care and ensuring vaccine safety.
